# Polygyny without wealth: popularity in gift games predicts polygyny in BaYaka Pygmies

**DOI:** 10.1098/rsos.150054

**Published:** 2015-05-06

**Authors:** Nikhil Chaudhary, Gul Deniz Salali, James Thompson, Mark Dyble, Abigail Page, Daniel Smith, Ruth Mace, Andrea Bamberg Migliano

**Affiliations:** Department of Anthropology, University College London, London WC1H 0BW, UK

**Keywords:** polygyny, hunter–gatherers, social capital, genetic quality, social networks

## Abstract

The occurrence of polygynous marriage in hunter–gatherer societies, which do not accumulate wealth, remains largely unexplored since resource availability is dependent on male hunting capacity and limited by the lack of storage. Hunter–gatherer societies offer the greatest insight in to human evolution since they represent the majority of our species' evolutionary history. In order to elucidate the evolution of hunter–gatherer polygyny, we study marriage patterns of BaYaka Pygmies. We investigate (i) rates of polygyny among BaYaka hunter–gatherers; (ii) whether polygyny confers a fitness benefit to BaYaka men; (iii) in the absence of wealth inequalities, what are the alternative explanations for polygyny among the BaYaka. To understand the latter, we explore differences in phenotypic quality (height and strength), and social capital (popularity in gift games). We find polygynous men have increased reproductive fitness; and that social capital and popularity but not phenotypic quality might have been important mechanisms by which some male hunter–gatherers sustained polygynous marriages before the onset of agriculture and wealth accumulation.

## 2. Introduction

Before the advent of agriculture 12 000 years ago, humans lived as hunter–gatherers—this subsistence mode occupies more than 90% of our species' evolutionary history [1]. Throughout this period, humans lived in foraging societies characterized by high mobility and no accumulation of material resources [2,3]. Given the relative modernity of the Neolithic transition, deciphering the social structure and selective pressures experienced by hunter–gatherers is invaluable in understanding the suite of evolutionary adaptations possessed by humans today. One remaining question regarding human social structure is the evolution of marriage systems, which have been demonstrated to have knock-on effects for inheritance systems, parental investment and intra-sexual aggression within human societies [4–6]. Combined evidence from extant hunter–gatherers, phylogenetic reconstruction and archaeological remains suggests a predominantly monogamous/serially monogamous system in human origins, with polygyny potentially being prevalent at low levels [7–11].

Although differences in male reproductive success have been explored in some foraging populations, these studies have focused on variation in frequency of extra-marital affairs (see [12] for review). However, the occurrence of contemporaneous legitimate partnerships between multiple women and one man, i.e. polygynous marriage, within a hunter–gatherer context remains largely unexplored. In contrast to extra-marital affairs, women engaging in polygynous marriages are incurring the substantial cost of sharing a provider for themselves and their offspring; it is for this reason that polygynous marriage is a particularly interesting phenomenon.

The most common explanation for polygynous marriage employed by human behavioural ecologists is known as the female choice model—an adapted version of the polygyny threshold model [13,14]. The premise is that a female's fitness is determined by the access to resources her mate can offer her. Therefore, polygyny occurs in societies where there are large inequalities in male wealth because, evolutionarily speaking, females are better off becoming the second partner of a wealthy man than the first of a poor one. This explanation has been applied successfully in a large body of within and cross-cultural anthropological research on human polygyny [15–17]. However, it is only relevant to societies in which material wealth is accumulated such as industrialized, agriculturalist and pastoralist, not hunter–gatherers. In fact, there is suggestion that among the Ache, hunter–gatherer families with polygynous marriages operate a resource deficit and depend more than others on food sharing from other households [18], which makes large-scale polygyny seemingly unsustainable in hunter–gatherers that do not have storage. To elucidate the incidence of polygyny in hunter–gatherers, who do not accumulate material wealth or defend individual territories, we must search for alternative explanations.

We first explore the fitness outcomes of BaYaka polygyny. Although previous anthropological research consistently finds that polygynously married men achieve higher reproductive success, these findings are derived from societies that accumulate wealth, and thus some wealthy men are able to afford multiple families [7,16,19]. Given the lack of material resources in BaYaka subsistence, polygynous men may be inadequately equipped to support multiple families. Therefore, here polygynous marriage could instead represent a maladaptive behaviour resulting in increased offspring mortality and lower fitness. In order to address this, we test how marital status affects a man's number of living offspring.

We also explore other possible mechanisms that could facilitate the achievement of polygyny by a few hunter–gatherer men through examination of marriage practices of the BaYaka Pygmies. Women may engage in polygynous marriages because certain men are of a sufficiently high quality that the fitness benefits outweigh the costs of marrying an already married man. Here quality refers to any attributes possessed by a man that ultimately result in increased fitness for a woman marrying him. We investigate whether polygynous BaYaka men differ in quality from their non-polygynous counterparts across two dimensions—phenotypic quality measured by physical attributes of height and hand-grip strength, and social capital quality determined by economic gift games. Phenotypic quality may increase a man's mate value as it reflects genetic quality, which will be inherited by his offspring, thus increasing their viability in a strenuous environment with high mortality risk [20]. Previous studies have mixed results; however, researchers have found positive associations between number of marriages (including serial marriages) and height in Baka Pygmies from Cameroon [21], and strength in the Hadza [22]. Alternatively, in the absence of material wealth, social capital may be the resource that enables certain men to afford multiple families; anthropologists have highlighted the importance of wide social networks to buffer risk associated with hunter–gatherer subsistence [23].

We find polygynous men do have increased reproductive fitness relative to their monogamous peers; and that social capital, but not our measures of phenotypic quality, might have been an important mechanism by which some male hunter–gatherers sustained polygynous marriages before the onset of agriculture and wealth accumulation.

## 3. Methods

### 3.1 Study population

Our study uses data from the Mbendjele BaYaka, a subgroup of the BaYaka who speak Mbendjele language and whose residence spans across the forests of Congo and Central African Republic. BaYaka subsistence techniques include hunting, trapping, fishing, gathering and honey collecting. The BaYaka live in *langos*—multi-family camps consisting of a number of *fumas* (huts) in which nuclear families reside; camp size tends to vary from 10 to 60 individuals. They are predominantly serially monogamous like most African hunter–gatherers. Nevertheless, there are a notable proportion of men who are/have ever been married polygynously in our study sample (14.3%). This is a rate of men who achieve polygyny in their lifetime, which may overstate polygyny prevalence as compared to other ‘snapshot’ estimates which calculate the proportion of men/women married polygynously at one specific point in time. However, comparably high levels have been found in other BaYaka Pygmy groups—e.g. Aka [24], using the snapshot method. Such estimates are considerably higher than most well-studied foraging groups, e.g. 4% in Ache; 6% in Kung [6], but probably more representative of foraging societies on the whole, which have a mean male polygyny rate of approximately 14% [25]. When a man has multiple wives simultaneously, they usually reside in different camps, among which he divides his time. We use the term marriage, however it is noteworthy that there is no formal marriage institution. Partnerships are acknowledged by the community when a man and woman begin living in a *fuma* together. This is followed by a period of bride service by the husband for his new in-laws [26].

Our study population consists of 70 men, of whom 10 have been polygynous, from five BaYaka camps in the Likoula and Sangha regions of Congo's Ndoki Forest (see the electronic supplementary material, figure SI). Not all data were collected in each camp, e.g. gift games were only played in the final three camps we visited; additionally, some individuals were unable to participate—sample sizes for each analysis are indicated.

### 3.2 Data collection

#### 3.2.1 Measuring the influence of polygyny on male reproductive success

Reproductive histories were recorded from adult men and women. Individuals were asked to list all of their children and spouses, specifying whether they were dead or alive and which partner they conceived each child with. If a man had more than one spouse, he was asked ‘when you started with the second woman, had you already finished with the first, or did you carry on with two women at the same time?’. A man is considered to be polygynous if the answer to this question is that he continued relationships with two women simultaneously. Therefore, men who have ever been polygynous are coded as polygynous even though they may not necessarily be so currently.

#### 3.2.2 Quantifying phenotypic quality

We inspect differences in two physical attributes, specifically hand-grip strength and height. Hand-grip strength was measured using a manual dynamometer. Participants had three attempts with each hand and were instructed to keep their arm straight and perpendicular to the ground. Height was measured using a Harpenden anthropometer. To ensure accuracy, two researchers would take the measurements, with one ensuring that the anthropometer was perpendicular to the ground and that the participant was standing straight, while the other noted the reading.

#### 3.2.3 Quantifying social capital

To measure social capital, we used the Gift Game procedure described in Apicella *et al.* [27]. In this game, participants were asked in private to nominate recipient(s) of three honey sticks. It was explained that participants could allocate the three honey sticks freely, i.e. give one stick to three different individuals or three sticks to one individual, etc. Recipients were permitted to nominate any individual from their camp other than him/herself. This game was played with all adults in the camp. In-degree in the gift game is used as a proxy for social capital, i.e. the more honey sticks an individual receives from other members of his camp, the greater his social capital.

#### 3.2.4 Age

In order to analyse whether the polygynous men in our sample had higher reproductive success, it is necessary to control for age. However, none of the individuals in our sample knew their own age, thus we had to create a relative age list and used age rank as a control (see the electronic supplementary material for details).

## 4. Analyses

(1) To test whether polygynous men have higher reproductive success we use multiple regression. The response variable is number of living children, with polygynous status (binary) as a predictor and log age rank as a control. Log age rank is used since this fits our data better than age rank squared.
(2) To determine whether phenotypic quality/social capital explains why certain men are polygynous, we conduct one-way randomization tests with 9999 Monte Carlo re-samplings comparing polygynous and non-polygynous men across these dimensions. For phenotypic quality, we compare their height and hand-grip strength (highest score from all attempts). For social capital, a comparison of *z*-scores for gift game in degree is used; it is necessary to create *z*-scores to control for camp size.

All analyses were conducted using R i386 3.1.1; we use the coin package for randomization tests.

## 5. Results

### 5.1 Men in polygynous marriages have higher reproductive success

**Figure 1.**
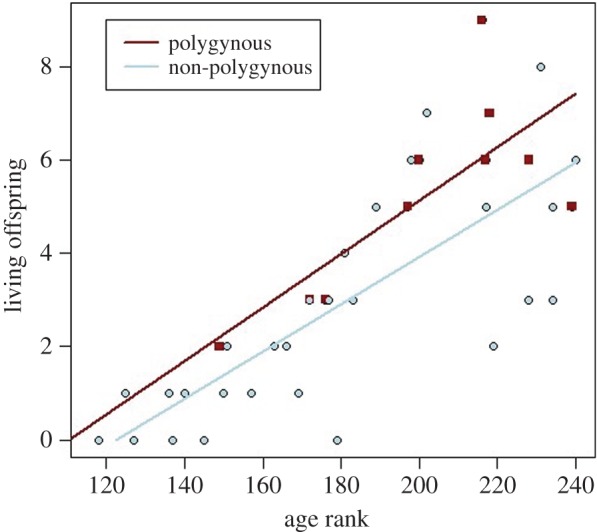
Scatter plot and regression lines of number of currently living offspring by age rank. Purple squares/line are polygynous individuals and blue circles/line are non-polygynous individuals.

Our results demonstrate that polygynously married men do have more living offspring for their age than men who are not polygynous (table 1 and figure 1).

**Table 1.** Multiple regression of number of living offspring on marital status, controlling for age rank. The predictor ‘polygynous’ is a dummy variable—its coefficient represents the change in number of living offspring for age when a man is coded as polygynously married. Thirty-nine refers to total sample size, of which 10 men were polygynous.

*n*=39, 10 polygynous	coeff.	*p*-value
polygynous	1.307	0.025*
log age rank	8.721	0.000***

***p<0.001, *p<0.05.

### 5.2 Phenotypic quality is not associated with polygyny

Our results do not provide support for the hypothesis that men who achieve polygyny are of higher phenotypic quality. Polygynous men in our sample are slightly taller and stronger than non-polygynous men, but these results are not significant (table 2*a*).

**Table 2.** One-way randomization tests comparing phenotypic quality and social capital of polygynous and non-polygynous men. Sample sizes are indicated: the first value is total sample size and the value in parentheses refers to the number of polygynous men in the sample.

	*n*	mean (polygynous)	mean (non-polygynous)	*z*	*p*-value
(*a*) phenotypic quality					
height (cm)	66 (10)	155.7	154.6	−0.500	0.616
hand-grip	62 (10)	46.6	45.7	−0.174	0.871
(*b*) social capital					
Gift Game *z*-score	44 (5)	0.842	−0.102	−2.015	0.034*

*p<0.05.

### 5.3 Greater social capital is associated with polygyny

**Figure 2.**
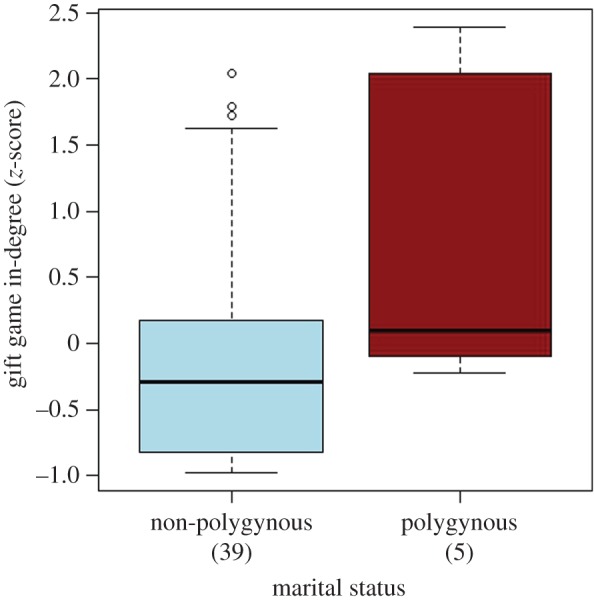
Boxplot comparing gift game in-degree *z*-scores of polygynous and non-polygynous men. *Z*-scores are standardized by camp, to allow simultaneous comparison of social capital of men from all camps by controlling for camp size. Hollow circles represent outliers. Sample sizes are indicated in parentheses.

Polygynous men have significantly more social capital than non-polygynous men (*p*=0.034); see figure 2 and table 2*b* for full results. In two of the three camps where the gift game was played, the individual with the highest number of gifts was polygynous. Both of these men were the *kombetis* (an appointed spokesperson for a camp) of their respective camps. In Longa, this individual received nine honey sticks compared to a camp male average of 2.6; similarly, in Ibamba, these figures are 17 and 4.5, respectively. In Ibamba, there were three polygynous men, who ranked first, third and fourth in popularity out of 12 men in that camp.

## 6. Discussion

The fact that polygyny rates correlate with wealth inequality in most human populations [5,17] raises the question of whether polygyny was even possible before the Neolithic transition, and whether human origins are strictly monogamous. Here we present a preliminary insight in to this question by exploring both whether polygynous marriage is actually beneficial to men in a hunter–gatherer context, and how certain men achieve polygyny without material wealth. We find polygynous men have greater reproductive success; and differences in our measures of social capital but not phenotypic quality explain which men achieve polygyny.

It is possible that polygynous marriage is a recent maladaptation among BaYaka men as a result of copying a Bantu pattern of marriage. However, even if higher rates of polygyny are new to the BaYaka Pygmies, they do not seem to be maladaptive for BaYaka men. Polygynous men do experience greater reproductive success in spite of the lack of accumulation of material wealth—they have more living offspring for their age than their non-polygynous counterparts.

With respect to the determinants of which men achieve polygyny, we assessed the importance of phenotypic quality and social capital. Strength and height have been frequently found to increase male attractiveness since they are signals of genetic quality [28–30]. In environments of high pathogen stress, such as those experienced by the BaYaka, women may place more value on genetic quality in order to increase the viability of their offspring [19,31]. Additionally, in contexts where male provisioning is less important, women shift mate selection strategies away from ‘resource shopping’ towards ‘gene shopping’ [9], and male signalling of quality becomes more fundamental in mating dynamics [32]. In contrast to a more a typical pattern among forager groups where the majority of provisioning comes from men's hunting production [33], among BaYaka Pygmies (Aka) from Central African Republic male and female contribution to subsistence is roughly equal in terms of calories [34]; herefore, we might expect BaYaka women to place relatively more value on genetic quality. In spite of this socio-ecological context, our results suggest that polygynous men do not differ significantly in strength or size. Here, we only examine two physical attributes; a recent study on the Hadza with numerous measures found effects on reproductive success that differed in direction and significance [22], highlighting the difficulty in operationalizing phenotypic quality with few variables. Thus, one must be cautious when generalizing these results. Additionally, the short stature of Pygmy groups may be a by-product of other positively selected life-history processes [35], and therefore individual variation in height may not be reflective of differences in phenotypic quality in this population.

The relative importance of social capital or phenotypic quality is also likely to be affected by BaYaka food sharing patterns. A variety of sharing systems have been identified within hunter–gatherer societies, in particular demand sharing and reciprocity [36,37]. With respect to mate value, if demand sharing is the predominant driver of food transfer, then food sharing is completely unbiased and widespread; therefore, a man's provisioning ability is less important, in turn raising the relative importance of his physical attractiveness. Conversely, under a system of reciprocity food transfer is not unbiased; rather, it reflects long-standing sharing relationships. A meta-analysis of human and non-human primate food sharing highlights that reciprocal transfers are more prevalent in the Central African Republic BaYaka (Aka) than any other group included in the study [38]. Establishing sharing relationships is likely to be crucial to securing a stable nutritional income for one's family; hence, as shown in our results, we expect that social capital is likely to be a central component of a BaYaka man's mate value—more so than in other human societies.

We find polygynous men have significantly more social capital than their non-polygynous counterparts. This finding is unlikely to be due to reverse causality, i.e. polygynous men having more affinal kin playing the game, since none of the polygynous men in our sample had multiple wives living in the same camp.

We can only speculate about how social capital assists men in obtaining and supporting multiple reproductive partners at the same time. One possible pathway may be that men with a large social network are more effectively able to buffer food risk. Owing to the absence of food storage in the dietary niche occupied by humans for the majority of our evolutionary history, risk reduction is considered to have been one of the most important adaptive problems faced by our species and the foundation of our sociality [39]. This remains the case for modern day hunter–gatherers, and thus within these communities it is possible that individuals who have more social capital can overcome this adaptive problem more successfully via widespread food sharing networks. These individuals with an abundance of social support may be more attractive marital partners, and the only ones capable of supporting multiple families. Perhaps ensuring bias in food sharing is how social capital translates into the acquisition of multiple wives.

When a polygynous man is staying in another camp with a different wife, his foraging contribution is completely absent. Thus, unless female production covers 100% of provisioning, women incur a cost by marrying polygynously. Moreover, in this group of BaYaka only men hunt, and the protein and fat content of meat they provide are necessary dietary complements to female gathering. Additionally, fathers in BaYaka groups have been found to provide more direct care than any other society in the world [34]. It is this paternal care that facilitates female production, freeing up mothers to invest time in foraging. Thus, a polygynously married woman, in the absence of her husband, is also likely to encounter more difficulties balancing the trade-off between direct care and foraging. Thus, the wives of polygynous men may rely on their husband's large social network for provisioning and allocare when he is residing in another camp with a different wife.

BaYaka camps have a political position of *kombeti*, which can be described as an appointed spokesperson who has influence, but not absolute authority, over camp decisions regarding subsistence and movement, as well as interactions with farmer and other non-BaYaka groups [24]. There are numerous reasons why these individuals may have higher mate value to women. Although the BaYaka generally do not accumulate material resources, they occasionally receive resources such as money, clothes, machetes, etc., from interaction with tourists, researchers, farmers and government social programmes. *Kombetis* may manipulate the distribution of these resources—in the past when we have given gifts for a camp, the *kombeti* would direct their distribution and usually end up with a larger share (not necessarily overtly); they also receive more goods such as cigarettes from farmers [34]. It is noteworthy that this only occurs with resources that come from outside groups; *kombetis* have no authority over resources produced by camp members themselves. Additionally, these men, through their prestige, may be more able to influence group decisions in their favour thus increasing their mate value further; such an effect has been found in prestigious Tsimane men who in turn have favourable fitness outcomes [40]. Dental research also suggests *kombetis* may have access to a more nutritious diet, and this may be a result of other camp members sharing more high-quality foods with them [41]. This position of kombeti is appointed, thus attainting and maintaining this status relies on social capital, and not excessively exploiting it. In our sample, there are only two *kombetis*, both of whom are polygynous and had the most social capital in their respective camps, providing some support for this pathway; confirmation would require conducting pathway analysis with a larger sample size.

This research attempted to identify the determinants and outcomes of polygynous marriage within a society that lacks material wealth. Our findings that polygynous hunter–gatherer men experience advantageous fitness outcomes and have more social capital provide an important step in understanding hunter–gatherer marriage, and whether/how polygynous marriage was even possible before the Neolithic. Some areas of the world like Australia are notorious for high levels of polygyny among hunter–gatherers [42], and cross-cultural research indicates that on average approximately 14% of men are polygynous in foraging groups [25]. Understanding how such systems evolved in spite of unpredictable hunting returns and the need for provisioning has always been a challenge.

To enhance our understanding of this topic further, it would be interesting to investigate why certain men have more social capital than others, and how men compete across this dimension. Australian Aborigine men enhance their status via initiation rites and secret knowledge [42]; perhaps a similar process is occurring with the BaYaka. There are a vast number of initiation rites that occur at different stages of a man's life, some which all men participate in and others which only a fraction of men undergo. Status can be further augmented by becoming a *konja wa mokondi*, where one becomes an authoritative figure in the initiation of others. *Nganga* is another of the few recognized positions held by the BaYaka and refers to healers with advanced knowledge of *bwanga*—forest medicines [24,26]. The attainment of such positions relies on specialist knowledge and individuals who bear such knowledge are perceived to provide benefits to the group, and in turn are likely to accrue social capital. Additionally, in many foraging societies good hunters have high social status, which in turn provides benefits of extra-marital affairs and favourable treatment from camp members who value their contribution and quality [12,43]. Another remaining question is why women choose to enter into polygynous marriages with popular men. Potential starting points to address this question include examining whether men with more social capital have advantageous food sharing networks; or whether social network size is a predictor of becoming *kombeti*, and the extent to which this position facilitates the manipulation of communal resources.

## Supplementary Material

Supplementary Material - Information on contructing age rankings and location of camps.

## Data accessibility

The data supporting this article are available in the Dryad Digital Repository (doi:10.5061/dryad.0dk3n).
