# Supplementary material for: Polygyny without wealth: popularity in gift games predicts polygyny in BaYaka Pygmies
Source: R Soc Open Sci. 2015 May 6;2(5):150054. doi: 10.1098/rsos.150054 (PMC4453254; doi:10.1098/rsos.150054)
Supplement: Supplementary Material - Information on contructing age rankings and location of camps. [file rsos150054supp1.docx]

**Polygyny without Wealth: Popularity in gift games predicts polygyny in BaYaka Pygmies**

**Supplementary Material**

**Constructing a Relative Age List**

In order to create a relative age list we took photos of all the members of camps Longa, Enoko and Mbaya. These were then divided in to rough age cohorts based on visual estimations of the team and our translators/guides. Then we asked all adults of the largest camp (Longa) to sit down around a mat upon which we would lay out all the photos of a given cohort. Firstly, we would ask which individual in the cohort was the oldest, and then pick up all the remaining photos. Then one by one we would lay down the photos, and ask the respondents the name of the individual to ensure they knew who it was. Next we asked where the photo should be positioned – to the right of a placed photo if the individual on the current photo was older, and to the left if s/he was younger. After each placement we confirmed that the newly placed photo was older than the photo on its left and younger than that on its right; in some instances where individuals were considered equal in age, their photos would be placed above/below each other. If there was any disagreement, participants were told that they must discuss amongst themselves and arrive at one answer together. Cohorts were added to the mat one by one. After ranking within a cohort had finished, using the same process we checked whether individuals judged to be oldest/youngest of that cohort were older/younger than those on the edges of the adjacent cohorts. Regular breaks were taken to avoid participants losing concentration.

Masia was the final camp we visited after a break in the logging town. This camp was partially constituted of members of the first 3 camps and some new individuals. Therefore, we presented the initial ranking provided by members of Longa and asked the members of Masia communally where each of them fitted in.

Ibamba is the most isolated camp, and the only of the five camps which does not lie on the same logging road that connects the other four. The Mbendjele in this camp were not familiar with members of the other camps, and vice versa. Therefore, using the same method we created a distinct relative age list for this camp. We then merged this ranking with the large ranking of the other four camps. The large ranking included 240 individuals, compared to the Ibamba ranking which included only 60. Thus assuming an equal population-age structure, we multiplied all rankings of individuals from Ibamba by four to give them an appropriate position in the larger ranking. Although not ideal, this method appears to be sufficient when cross-checking. For instance the oldest ranked child in Ibamba who had not yet completed his dental development i.e. according to his teeth he was 11-12 years of age, was ranked 20; hence his counterpart in the large ranking would be of rank 80. Indeed, the individual ranked 80 in the large ranking fell in to this same one year wide category of dental development. Similarly, in Ibamba the youngest married woman was ranked 28 an equivalent of rank 112 in the large ranking. In the large ranking the youngest married woman was rank 115, again suggesting the assumption of an equivalent population-age structure is adequately robust. Note only three men from this camp were used in the age fertility analysis, as data on the non-polygynous men was not collected in this camp.

Thus far we have been unable to translate this final relative ranking in to absolute ages since none of our study population know their age, therefore we lack any 'anchoring' points within our ranking.

**Locations of Camps**


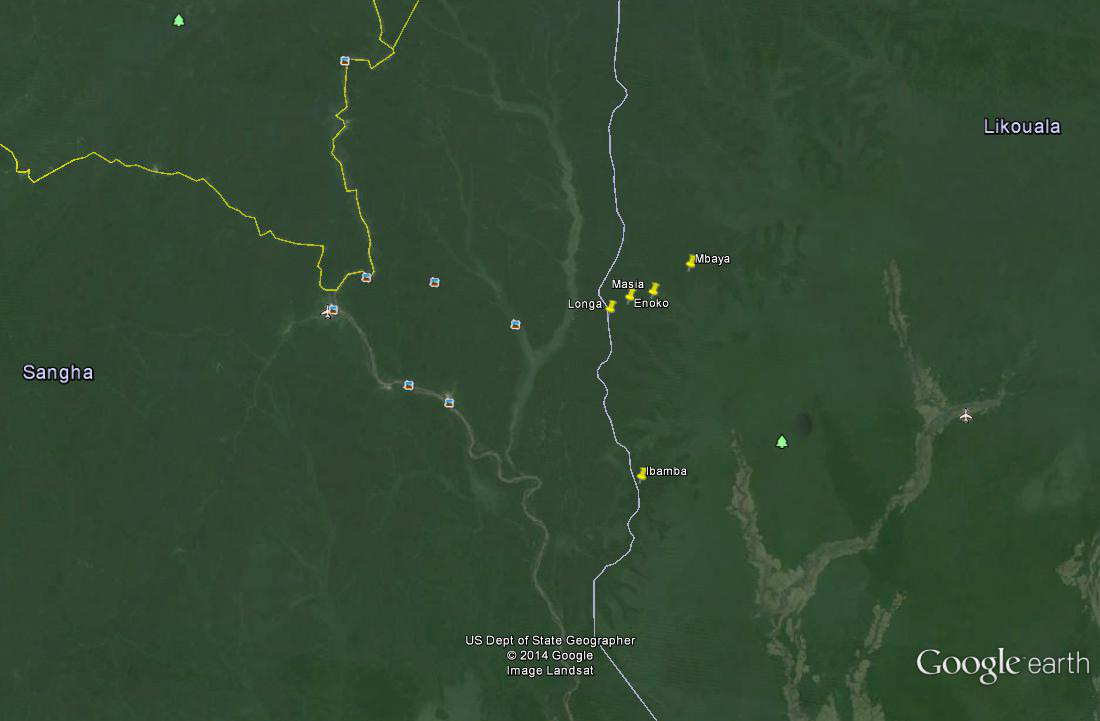
Our study sample included men from five different camps - Longa, Enoko, Mbaya, Masia and Ibamba. The first four of these lie by the same logging road connecting Pokola (the logging town) and Minganga (a farmer village); whereas Ibamba is on a different road south of the other camps. See Figure S1 below for map.

Figure S1: Map indicating location of the five camps which constitute the study sample. Map was generated using GPS co-ordinates in Google Earth.
